# Managing Medical Emergencies With Palliative Medicine in a Patient With Metastatic Breast Carcinoma Receiving Only Supportive Care

**DOI:** 10.7759/cureus.80619

**Published:** 2025-03-15

**Authors:** Parth S Jha, Prasun P, Srikanta Panda, Jayita K Deodhar

**Affiliations:** 1 Palliative Medicine, Tata Memorial Hospital, Tata Memorial Center, Homi Bhabha National Institute, Mumbai, IND; 2 Palliative Medicine, Advanced Centre for Treatment, Research and Education in Cancer (ACTREC) Tata Memorial Center, Mumbai, IND

**Keywords:** best supportive care, diabetic ketoacidosis, emergency palliative care, metastatic breast carcinoma, palliative care, palliative medicine, sepsis palliative care

## Abstract

We report the case of a 56-year-old female patient with synchronous breast carcinoma on supportive care alone, who presented to the emergency department with acute medical emergencies such as altered sensorium, anasarca, vesicular skin lesions, and hemodynamic instability. Initial investigations revealed a myriad of life-threatening conditions, including suspected brain metastasis, diabetic ketoacidosis, acute kidney injury and sepsis. Balancing symptom control with appropriate medical interventions while respecting the goals of care remains a critical challenge. Emergency management involved intravenous hydration, insulin therapy, empirical antibiotics, and symptomatic control with fentanyl and haloperidol. Throughout hospitalization, she received targeted antibiotic therapy, albumin infusions, and antiepileptics. She was gradually stabilized. Despite the complexity of her condition, she responded well to the instituted measures and was discharged in a stable condition after 14 days. This case highlights key principles in palliative emergency management, including the integration of medical knowledge to address overlapping critical illnesses. This holistic approach prioritizes symptom relief without futile escalation of care and includes effective communication with family members to align treatment with the goals of care.

## Introduction

Palliative care often presents unique challenges, particularly when patients with advanced malignancies experience acute medical emergencies. Managing emergencies in palliative care is a double-edged sword. While excessive intervention can dramatically decrease quality of life, suboptimal management can lead to imminent demise. It is also complicated by simultaneous management of multiple complex emergencies in the presence of several comorbidities and poor performance status. In such cases, integrating supportive care with timely medical interventions is crucial for managing life-threatening complications while aligning them with the patient’s goals of care [1]. This case report examines the management of a 56-year-old female patient with synchronous breast carcinoma who was on supportive care and presented to the emergency department with multiple critical conditions, including altered sensorium, anasarca, vesicular lesions, and hemodynamic instability. The patient’s complex clinical picture highlighted several key aspects of palliative emergency care such as the management of diabetic ketoacidosis (DKA) in terminally-ill patients, the identification and treatment of infections, pain and delirium management, supportive care, and the role of effective communication with family members. In the face of overlapping critical illnesses, timely intervention and symptom relief were prioritized, ensuring that medical care was appropriate and aligned with the patient’s prognosis. This patient's case emphasized the importance of a holistic approach in palliative care, addressing both physical and emotional needs, while making careful decisions regarding the escalation or limitation of care. This case also draws attention to the need for personalized care plans that respect the patient’s wishes, demonstrating how proactive management and clear communication can improve outcomes even in patients with advanced cancer. Early discussions about goals of care lead to a better quality of life, ensure care is consistent with the goals, reduce nonbeneficial medical care near death, ensure positive family outcomes, and lower costs. This makes such communication a necessary part of clinical care [2].

## Case presentation

**Figure 1:**
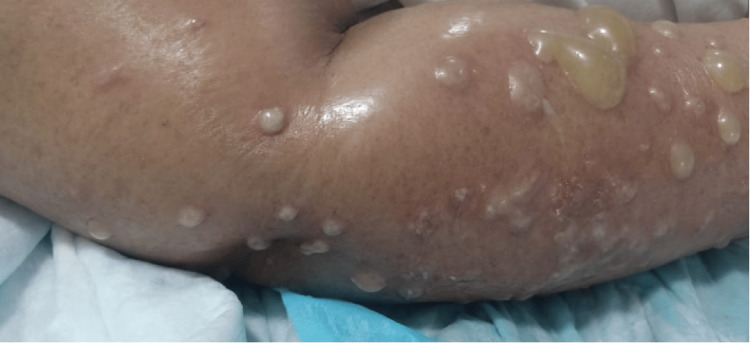
Lesions on the patient's right upper limb

A 56-year-old female patient with a known history of synchronous breast carcinoma was on supportive care alone. She was also suffering from type 2 diabetes mellitus (DM) for the past seven years and was managed with metformin 500 mg twice daily. She presented to the emergency department at night with altered sensorium, decreased urine output, generalized edema, and vesicular lesions over the right upper limb (Figure 1).

On presentation, her blood pressure was 90/60 mmHg (normal value: 120/80 mmHg) and pulse rate was 120 beats/min (normal value: 60-100 beats/min). On examination, she had anasarca, multiple vesicular lesions on the right upper limb with surrounding inflammation, and grade three bilateral pitting pedal edema. Her Richmond Agitation Sedation Scale (RASS) on presentation was +2. Investigations revealed a random blood sugar of 421 mg/dL (normal value: <200 mg/dL) and urine ketones of 3+ (normal value: none detected). This constellation of suspected brain metastasis, DKA, acute kidney injury, and sepsis required compelling and complex emergency management. Complete blood profile, renal function tests, liver function tests, serum electrolytes, arterial blood gas, and blood culture were carried out. Pus culture was also done from the open fluid vesicle.

Intravenous (IV) hydration with normal saline at 200 ml/hour, IV bicarbonate, and regular insulin were given for managing the DKA. Empirical antibiotics i.e., cefoperazone along with sulbactum, were initiated. The background pain was managed with IV fentanyl and paracetamol with doses as needed. The delirium was managed with IV haloperidol. After seven hours of ongoing management, the patient regained orientation to time, place, and person.

Over the course in the hospital ward, she received a complete antibiotic course, albumin infusions, and absorbable dressings. The pus culture report implied *Pseudomonas aeruginosa*, for which piperacillin and tazobactum was given according to the sensitivity report. She had one focal seizure after five days for which antiepileptics were started. She also required one packed cell volume transfusion for anemia of chronic disease. After 14 days of hospital treatment, she was discharged in a stable condition.

Throughout the treatment, her primary caretakers (her son and husband) were informed about every step of the treatment and its rationale. They were aware of the poor prognosis and the non-feasibility of disease-directed treatment. All the comfort measures and the appropriate medical measures taken to address what was correctable, without admission to intensive care unit, were discussed with the family.

## Discussion

A palliative care emergency is a rapid and serious decline in a patient's condition that demands immediate medical attention to prevent severe health consequences and a diminished quality of life. Such emergencies typically stem from complications of advanced illnesses and are more commonly observed in individuals with progressive cancer or other life-limiting conditions [1]. We explored the critical role of managing multiple complex emergencies in palliative medicine, underscoring the need for an integrated approach that leverages in-depth general medical knowledge. Palliative care often involves addressing multifaceted symptoms and psychosocial issues, requiring practitioners to navigate complex interactions between various medical conditions.

Initial stabilization of the present case included management of DKA. DM is significantly more common in patients with cancer, occurring at nearly six times the rate seen in the general population. Studies indicate that DM contributes to higher mortality in this group. Managing DKA in terminally-ill patients is particularly challenging, as its symptoms can mimic those at an end-of-life decline. We started the patient on IV hydration with normal saline at 200 mL/hour, regular insulin, and IV bicarbonate. Despite the lack of standardized guidelines for management of DM in hospice care, timely treatment of DKA may help prolong the lives of patients [3].

A study conducted in China found that the primary trigger for DKA was infection, accounting for 40.1% of cases, followed by discontinuation of hypoglycemic therapy at 16.8% and unexplained causes at 36.9% [4]. There was no history of hypoglycemic therapy discontinuation in our patient. Infection, the leading precipitant of DKA, is strongly associated with an increased risk of mortality [5]. In our patient, the pus culture revealed the presence of *Pseudomonas aeruginosa*, for which piperacillin-tazobactam was administered based on the sensitivity report. During her hospital stay, she completed the full course of antibiotics and received absorbent dressings for wound management. Timely and accurate identification of infectious sources allows for appropriate antibiotic administration, which improves the patient's condition and also helps prevent the development of antimicrobial resistance [6].

Proactively addressing symptoms in patients with severe life-limiting or terminal illnesses can enhance their quality of life, as well as that of their loved ones, regardless of how much time they have left [7]. Pain is the most dreaded symptom for individuals with cancer and other terminal illnesses. Opioids are the primary medications used to manage moderate to severe pain in these patients [8]. Background pain in our patient was managed with IV fentanyl and paracetamol with doses as needed.

Delirium is common in cancer patients, especially in those with advanced disease or high vulnerability. Older age, a lower performance status, brain metastasis, urinary tract infection, sepsis, hyponatremia, and hypercalcemia are independent factors associated with delirium [9]. It creates distress and challenges for both patients and healthcare providers in managing agitation and assessing symptoms. Treatment depends on prognosis and care goals, with antipsychotics typically used for symptom control, and some patients who are refractory requiring sedation [10]. Various screening and monitoring tools are available to manage the same. One such screening tool in standard practice is the RASS which has shown excellent interrater agreement [11]. In our patient, the RASS score was +2 at presentation, leading to treatment with IV haloperidol.

The patient was diagnosed with multiple underlying conditions requiring comprehensive management. To address the complications, additional supportive measures were implemented. These included albumin infusions to manage hypoalbuminemia and anasarca, a packed cell volume transfusion to treat anemia, and anticonvulsants for the management of a post-focal seizure. These interventions were essential in stabilizing the patient’s condition and preventing further complications.

Palliative symptom management adopts a holistic approach, focusing on alleviating not only physical symptoms but also addressing psychological, social, and spiritual distress to provide comprehensive relief [7]. This specific case report aims to underscore the importance of a holistic, multiplenary strategy that not only alleviates suffering but also enhances the quality of life for patients and their families. Understanding the care goals of the patients in the context of serious illness is crucial for providing high-quality care that aligns with their priorities. Early discussions about these goals can lead to better quality of life, reduced nonbeneficial medical care near death, improved care consistent with the goals, positive family outcomes, and lower costs. Such communication thus becomes a necessary part of clinical care [2]. While it was the family’s wish to do everything possible to prolong her life and decrease her suffering, and correcting the correctable, they also understood the futility of escalating treatment to admission in the intensive care unit. They consistently sought to remain by her side throughout the duration of her treatment.

## Conclusions

This case highlights the complexities of managing multiple coexisting emergencies in palliative medicine and the necessity of a multidisciplinary approach to care. Effective symptom control, timely intervention for acute complications, and addressing psychosocial concerns are critical in enhancing both patient comfort and overall quality of life. The successful management of DKA, infection, pain, and delirium in this patient underscores the importance of individualized and goal-oriented care. While aggressive treatments may not always be appropriate in terminally-ill patients, correcting reversible conditions can improve outcomes and provide meaningful time with loved ones. Early and continuous discussions about the goals of care remain essential in ensuring that medical decisions align with the patient's and the family's preferences, ultimately fostering a compassionate and dignified end-of-life experience.
